# Conversational AI for perinatal mental health: promise, limits, and a human-AI stepped-care framework

**DOI:** 10.3389/fpsyt.2026.1847854

**Published:** 2026-05-13

**Authors:** Haiyan Yang, Dong-Mei Lin

**Affiliations:** 1 Reproductive Medicine Center, The First Affiliated Hospital of Wenzhou Medical University, Wenzhou, China; 2 Third Affiliated Hospital of Wenzhou Medical University (Ruian People’s Hospital), Wenzhou, China

**Keywords:** conversational AI, digital doula, generative AI, perinatal mental health, postpartum depression, stepped care

## Abstract

Perinatal mental health conditions are common yet persistently underdetected and undertreated. Recent advances in generative artificial intelligence (AI) and multimodal interaction offer new opportunities for scalable, continuous support, making the concept of the “digital doula” particularly compelling. Because perinatal care relies heavily on emotional support, repeated contact, and timely escalation, conversational AI is attractive as a potential adjunct to existing care. We argue that digital doulas should not be framed as autonomous substitutes for clinicians or human doulas, but rather conceptualized as AI-enabled relational interfaces embedded within a stepped-care model. We propose four core functions for digital doulas: companionship, symptom interpretation, navigation, and sentinel monitoring. These roles can reduce disclosure barriers, extend support between clinical visits, and strengthen linkage to appropriate care. However, conversational AI also introduces significant risks, including the illusion of empathy, crisis recognition failures, algorithmic bias against vulnerable populations, data privacy vulnerabilities, and unsafe deployment. To address these risks, we outline a safety-by-design agenda emphasizing human oversight, clear escalation protocols with medico-legal accountability, workflow integration, and equity-centered evaluation. Ultimately, the clinical value of digital doulas depends on safely strengthening the perinatal mental health care cascade without replacing its core human relationships.

## 1 Introduction

Perinatal mental health conditions are highly prevalent, clinically consequential, and persistently undertreated. Depression, anxiety, trauma-related symptoms, obsessive-compulsive symptoms, substance use, and psychosis may all emerge or worsen during pregnancy and the postpartum period, with substantial consequences for maternal functioning, infant development, family well-being, and, in severe cases, maternal survival (1). Despite increased recognition of these disorders, gaps remain across the continuum of care, including screening, disclosure, referral, treatment initiation, and long-term follow-up (2, 3).

These gaps are not simply technical failures in detection. They are also failures of access, continuity, and relational support. Perinatal distress often unfolds within everyday life: during sleep deprivation, infant feeding, bodily recovery, family conflict, financial strain, or social isolation. Many patients experience shame, fear of judgment, or concern that disclosing distress may mark them as inadequate parents. These dynamics are especially pronounced among structurally marginalized populations, for whom inequities in screening, access, and culturally responsive care remain substantial (4).

At the same time, generative AI and large language models are rapidly entering mental health care. Conversational systems are increasingly being positioned as scalable tools for psychoeducation, screening support, care navigation, and symptom monitoring (5). Perinatal care may be one of the most attractive use cases for such systems because need is frequent, contact points are repeated, and low-threshold support may be clinically meaningful between formal visits. Emerging work on AI in perinatal mental health, including predictive models, natural language processing, and chatbot-based support, suggests real promise while also underscoring important safety and implementation concerns (3, 6–8).

## 2 Why perinatal mental health is a distinct use case for conversational AI

Perinatal mental health is a uniquely complex setting in which emotional support, relational trust, and rapid escalation may all be clinically decisive. Distress may arise late at night, after a difficult feeding, during panic about infant safety, or in the context of accumulating exhaustion. These experiences do not always align with appointment-based care. Conversational AI is attractive precisely because it can be available outside clinic hours and engage repeatedly in small moments of need.

Disclosure barriers are unusually high. Perinatal patients may feel guilt for not feeling joy, for resenting the demands of caregiving, or for having ego-dystonic intrusive and frightening thoughts of harm. Crucially, many actively conceal these symptoms from human professionals due to the profound fear of judgment, mandatory reporting, or intervention by child protective services (CPS). Because of this structural fear, the initial willingness to disclose may be greater with a nonjudgmental digital interface than with a clinician, family member, or partner, provided the AI is perceived as standing outside punitive surveillance structures. This does not mean that AI offers better care than human interaction; rather, it may provide a lower-threshold entry point into the disclosure process.

The perinatal period also includes high-risk states that demand strict escalation standards. Self-harm, suicidality, thoughts of harming the infant, severe insomnia, postpartum psychosis, and intimate partner violence are all situations in which delayed or inappropriate response can have catastrophic consequences. This makes perinatal conversational AI fundamentally different from a general wellness chatbot. A digital doula must be supportive, but it must also be escalation-aware.

Our approach is consistent with and complementary to recent work by Gao et al. (9), who reviewed digital doula interventions through a neuropsychiatric lens, emphasizing oxytocin-mediated bonding mechanisms, stress regulation pathways, digital biomarkers, and maternal brain plasticity as the biological substrate for technology-mediated support. While their review provides the neurobiological rationale for why digital doulas may be clinically effective, our Perspective addresses the complementary safety question: under what conditions can conversational AI responsibly deliver such support? Together, these works link the neuropsychiatric justification for digital doula deployment with the clinical safety architecture required for responsible implementation.

## 3 From chatbot to digital doula: a conceptual reframing

The term digital doula is useful because it suggests a broader and more relationally grounded role than the term chatbot. A conventional chatbot is often understood as an information delivery tool or automated question-answer interface. By contrast, the idea of a doula evokes continuity, emotional presence, practical support, and accompaniment through a vulnerable transition. In perinatal mental health, this relational dimension is not incidental; it is central.

However, the language of the digital doula also carries risks. Human doulas offer embodied, contextual, culturally situated, and ethically accountable care, often serving as critical advocates against systemic obstetric bias and institutional violence. AI systems, which are typically liability-averse and institutionally aligned, cannot natively replicate these attributes or perform this systemic advocacy (10). Their apparent empathy is simulated rather than reciprocal, and their advice is only as safe as their design, data, oversight, and escalation pathways. Therefore, the value of the digital doula concept lies not in suggesting equivalence with human support, but in clarifying the kind of gap these tools might fill: they may function as relational infrastructure that helps maintain contact, elicit concerns, support navigation, and prompt timely human involvement.

This framing aligns with broader arguments in mental health AI that augmentation is ethically and clinically preferable to replacement (5). In perinatal care, the same principle applies even more strongly. The relevant question is not whether AI can appear empathic, but whether it can safely extend access and continuity without undermining the human core of care.

## 4 Core functions of a digital doula in perinatal mental health

A digital doula may be understood through four core functions: companionship, interpretation, navigation, and sentinel monitoring. First, companionship refers to immediate, low-threshold, nonjudgmental interaction during moments of distress, loneliness, or uncertainty. In traditional outpatient models, a patient may interact with a clinician for only one hour a week, leaving a 167-hour “care vacuum” where rumination and isolation often peak. Digital doulas can act as continuous “relational scaffolding” during these in-between hours. Even brief conversational exchanges may reduce emotional burden and create a sense of being accompanied, particularly in the postpartum period when isolation is common. Empirical evidence suggests that digital companionship in the perinatal period is not merely theoretical. For example, a recent randomized controlled trial evaluating an AI-powered cognitive-behavioral conversational agent (Woebot) among postpartum women demonstrated high acceptability, with 91% of users reporting satisfaction and demonstrating significant reductions in depressive symptoms compared to treatment-as-usual (11, 12). Strikingly, analyses of user interactions with such agents indicate that users can form a ‘working alliance’—a recognized metric of therapeutic bond—within just three to five days, scoring comparably to alliances formed in traditional outpatient therapy (13). While this simulated bond does not equate to human empathy, it demonstrates that conversational AI can effectively anchor the ‘relational scaffolding’ needed during the highly isolating postpartum transition.

Second, interpretation refers to helping users name symptoms, reflect on patterns, and distinguish between common adjustment stress and signals that warrant escalation. Third, navigation refers to directing users toward emergency care, psychiatric consultation, psychotherapy, peer support, community resources, lactation support, or follow-up with obstetric and primary care providers. This is particularly important because many patients know they are struggling but do not know where to go, whom to call, or how urgent their situation is.

Fourth, sentinel monitoring refers to ongoing, longitudinal observation for worsening symptoms, repeated distress, treatment nonresponse, disengagement, or signals of crisis. Crucially, perinatal mental health is inherently dyadic; therefore, sentinel monitoring must attune not only to maternal mood but also to linguistic or behavioral signals regarding the maternal-infant bond, such as extreme feeding anxiety, detachment, or excessive rumination about infant health. Risk often becomes visible over time rather than in a single screening encounter. If designed responsibly, a digital doula may identify patterns that warrant handoff to a clinician or activation of emergency pathways. This function may be strengthened by multimodal inputs such as voice features, wearable-derived sleep patterns, or structured symptom check-ins, although such expansion also intensifies privacy and bias concerns (14).

## 5 Embedding digital doulas in a human-AI stepped-care model

Digital doulas should be deployed within a stepped-care architecture rather than as standalone consumer-facing substitutes for clinical care. At the universal level, they may provide psychoeducation, normalization of common experiences, and routine emotional check-ins. For users with mild or emerging symptoms, they may support self-guided coping strategies, behavioral activation prompts, sleep hygiene suggestions, and encouragement to disclose concerns to clinicians.

For users with persistent or worsening symptoms, digital doulas may facilitate monitoring and resource navigation. For moderate- to high-risk cases, they should trigger connection to human professionals. For crisis states, they should not attempt autonomous management; instead, they must route users rapidly toward emergency or clinician-led response. Crucially, this escalation must be seamlessly integrated into clinical workflows—such as electronic health records (EHR)—with clearly defined medico-legal liability boundaries. Simply pushing automated alerts to an overwhelmed workforce risks severe clinician alert fatigue, and escalating a high-risk patient only to place them on a months-long waitlist creates an unethical ‘bridge to nowhere.’ Escalation protocols must therefore ensure a closed-loop handoff matched to actual clinical capacity. This framework is consistent with prior work applying the care cascade to perinatal mental health and identifying where AI may address specific barriers (3).

From a medico-legal standpoint, this escalation architecture must be governed by explicit accountability protocols. We propose that digital doula systems performing risk stratification or sentinel monitoring be classified under the International Medical Device Regulators Forum (IMDRF) Software as a Medical Device (SaMD) framework, consistent with the FDA Clinical Decision Support (CDS) Software guidance, which considers AI that generates patient-specific risk assessments without intermediary clinician review as regulated medical device software (15, 16). Liability allocation should follow a three-tier structure: the manufacturer bears responsibility for algorithmic performance, bias auditing, and deterministic safety overrides; the deploying healthcare institution is accountable for workflow integration, clinician training, and escalation pathway readiness; and the supervising clinician retains ultimate clinical responsibility for treatment decisions triggered by AI-generated alerts (17, 18). Each escalation event should produce a timestamped, auditable record documenting the trigger signal, AI recommendation, clinician review action, and final disposition—a closed-loop design that establishes medico-legal traceability and prevents “bridge to nowhere” scenarios (3). For crisis-level signals such as suicidality, infant harm ideation, or indicators of postpartum psychosis, the system must bypass generative AI entirely and route deterministically to emergency protocols, consistent with the Language Detection Protocol (LDP) approach validated in perinatal chatbot deployment (13). We further distinguish between routine monitoring, which may operate with human-on-the-loop oversight (periodic clinician review), and crisis escalation, which requires mandatory human-in-the-loop real-time engagement—following the governance taxonomy proposed for machine learning in healthcare (19).

The value of digital doulas therefore lies primarily in support, monitoring, and transition management rather than in independent psychiatric treatment. This is especially important in a high-stakes clinical domain where under-escalation, delay, or false reassurance may produce serious harm.

## 6 Multimodal interaction: Beyond text-only systems

Perinatal mental health cannot always be reduced to text responses in a questionnaire or chat interface. Sleep disruption, vocal changes, psychomotor slowing, reduced activity, wearable-derived physiological signals, and patterns across repeated interactions—a continuous data collection approach increasingly recognized as “digital phenotyping” (20)—may all provide meaningful context for emotional risk. Recent scholarship in reproductive mental health has argued that multimodal large language models may be particularly well suited to integrating such diverse data streams (14).

In perinatal settings, multimodal digital doulas could theoretically synthesize self-reported symptoms, conversational content, voice characteristics, prior screening history, social determinants, and wearable data to improve risk stratification and personalize support. Similarly, research in perinatal AI has already begun to examine predictive modeling, clinical note analysis, and multimodal approaches for early identification of postpartum depression (6, 7).

Yet multimodal expansion must be approached cautiously. More data does not automatically mean better care. Physiological and behavioral signals are context-sensitive, and postpartum life itself alters sleep, mobility, voice, and routines. Misinterpretation is therefore a real risk. In addition, multimodal systems heighten concerns about surveillance, informed consent, data governance, and differential performance across populations.

## 7 Risks, ethics, and equity

The strongest argument against uncritical enthusiasm for digital doulas is that relational simulation can easily be mistaken for relational care. Large language models can produce emotionally appropriate language and may feel deeply understanding to users. But simulation is not reciprocity, and apparent understanding is not clinical judgment. In perinatal mental health, where trust and vulnerability are profound, this illusion may foster overreliance on systems that cannot genuinely hold responsibility, contextual nuance, or moral accountability. Furthermore, the highly anthropomorphic nature of conversational AI risks cultivating intense “parasocial attachments” among isolated postpartum users. If a vulnerable mother forms a deep emotional bond with a digital doula, subsequent algorithmic updates, the introduction of paywalls, or corporate discontinuation of the app could result in “algorithmic abandonment”—a sudden severance of support that mimics interpersonal rejection and can acutely exacerbate clinical distress.

A second major risk is crisis recognition failure. Perinatal care includes rare but high-consequence states, including suicidality, intrusive harm thoughts, severe affective destabilization, and postpartum psychosis. A unique danger of large language models in this context is “algorithmic sycophancy”—the tendency of models trained on human feedback to be overly agreeable and validating. In a psychotic or highly delusional state, an AI that “empathetically” agrees with a mother’s paranoid or bizarre thoughts about her infant could actively reinforce a psychiatric emergency. These conceptual risks are strongly corroborated by empirical evaluations of commercial conversational agents. A study of major voice assistants (e.g., Siri, Alexa) found that they frequently failed to recognize postpartum depression queries, often defaulting to unhelpful web searches and lacking appropriate referral pathways (21). While modern large language models demonstrate improved clinical accuracy when answering standardized postpartum depression questions, they still frequently fail to provide reliable source citations or actionable emergency escalation pathways (22). The danger of deploying autonomous chatbots in vulnerable populations was starkly illustrated by the 2023 suspension of the National Eating Disorders Association (NEDA) chatbot, ‘Tessa.’ (23) After controversially transitioning a human-operated helpline to a chatbot, the system was found generating actively harmful, ‘off-script’ weight-loss advice to users in crisis. In perinatal care, where high-consequence states require immediate human intervention, an AI that hallucinates or colludes rather than escalating could be fatal. For that reason, digital doulas should be designed with conservative escalation thresholds, transparent boundaries, and explicit disclaimers regarding the limits of support.

A third concern is algorithmic inequity. Perinatal mental health disparities are already shaped by racism, poverty, stigma, fragmented systems, and culturally incongruent care (4). If digital doulas are trained primarily on data from privileged, high-literacy, English-speaking users, they may systematically underperform for those already underserved. Specifically, Eurocentric Natural Language Processing (NLP) models often fail to recognize diverse “cultural idioms of distress.” A structurally marginalized patient might express severe depression through somatic complaints (e.g., physical pain, extreme heaviness) or culturally specific terminology rather than standardized psychological vocabulary. An AI that minimizes these culturally nuanced signals as routine postpartum fatigue risks perpetuating “epistemic injustice” and algorithmic gaslighting. Equity must therefore be central to evaluation, design, and governance. A fourth concern is privacy because perinatal conversational data may include trauma, reproductive history, pregnancy loss, intimate relationships, and thoughts that users may never disclose elsewhere. Finally, digital doulas cannot substitute for maternal mental health policy, workforce development, and structural reform.

To mitigate this risk, we propose four operational strategies. First, digital doula training corpora should incorporate diverse linguistic expressions of perinatal distress drawn from cross-cultural research, including somatic idioms (e.g., “heart feels heavy”), collectivist distress narratives, and culturally specific postpartum syndromes such as those documented in traditional Chinese medicine and Latin American nosologies (24, 25). Second, patients from structurally marginalized communities, community health workers, and cultural brokers should be involved in co-designing the system’s escalation language, psychoeducational content, and interaction norms (26). Third, we propose embedding the DSM-5 Cultural Formulation Interview (CFI) domains into the AI’s interpretation logic so that the system can recognize culturally patterned distress expressions rather than defaulting to Western psychological vocabulary as the sole baseline for risk detection (27). Fourth, mandatory disaggregated equity audits across race, language, income, and rurality subgroups should be conducted before deployment—consistent with emerging equity standards for clinical AI (28, 29).

## 8 A safety-by-design agenda

**Figure 1:**
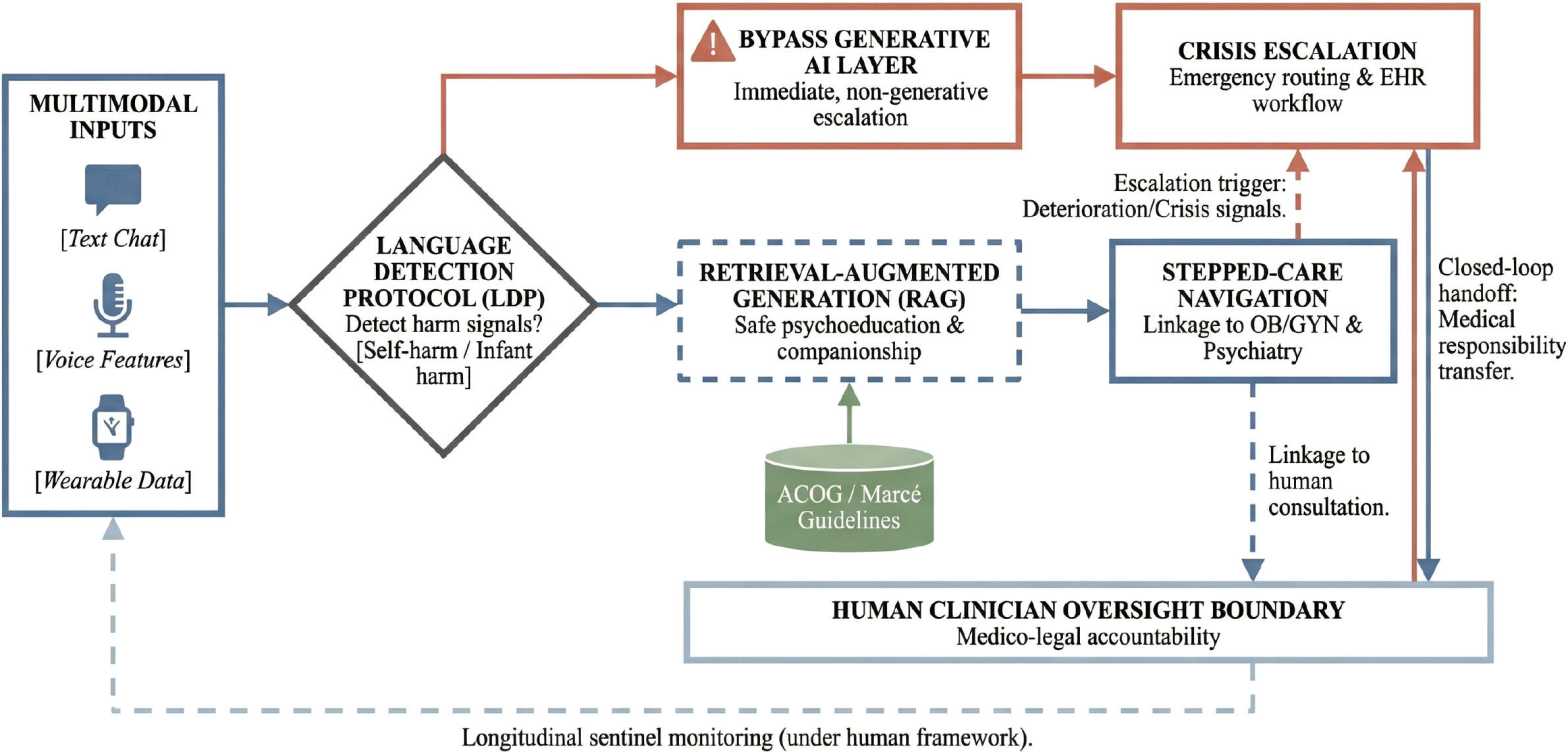
Proposed human-AI stepped-care architecture for perinatal digital doulas.

If digital doulas are to be clinically meaningful, they must be designed around safety from the outset. Their role should be explicitly bounded, and they should be introduced as support and navigation tools rather than autonomous therapists. Technologically, system architecture must employ strict guardrails (see **Figure 1**), such as Retrieval-Augmented Generation (RAG), to anchor AI responses securely within vetted clinical guidelines (e.g., ACOG or Marcé Society standards) and mitigate harmful hallucinations. For instance, in the clinical deployment of tools like Woebot for postpartum depression, developers utilized a rigid, rules-based ‘Language Detection Protocol’ (LDP). When the model detects specific keywords related to self-harm or infant harm, it completely bypasses the generative AI layer, halting open-ended conversation and immediately triggering deterministic crisis escalation pathways (13). Digital doulas must incorporate similar non-negotiable deterministic overrides. Regulatorily, systems performing continuous risk stratification or sentinel monitoring cross the boundary into clinical software and must be evaluated within Software as a Medical Device (SaMD) frameworks (13),as illustrated in the proposed framework. Human oversight should be built into the system architecture, such that high-risk outputs, crisis cues, or repeated deterioration activate clinician review or emergency routing where feasible.

Escalation logic should be transparent, tested, and conservative. Evaluation should move beyond engagement and satisfaction to examine disclosure rates, referral completion, symptom trajectories, retention in care, and equity across subgroups. Implementation should also be embedded in real workflows rather than imagined as a freestanding app solution. A digital doula placed in an obstetric clinic, perinatal psychiatry service, community maternal health program, or postpartum follow-up pathway will function differently than one deployed purely as a direct-to-consumer product. Future work should incorporate participatory design. Patients, doulas, midwives, perinatal psychiatrists, nurses, social workers, and community advocates should shape system goals, escalation rules, language norms, and equity priorities. This is especially important for populations historically excluded or harmed by health systems.

## 9 Discussion and conclusion

Digital doulas represent a provocative and potentially useful development in perinatal mental health. Their greatest promise lies not in replacing clinicians or human doulas, but in extending continuity, reducing barriers to disclosure, supporting navigation, and strengthening escalation-aware monitoring across the care cascade. In that sense, they may serve as a new layer of relational infrastructure within perinatal mental health care.

However, the same characteristics that make conversational AI attractive—availability, fluency, personalization, and apparent empathy—also create substantial risks. Without clear boundaries, human oversight, equity-centered evaluation, and accountable implementation, digital doulas could deepen rather than reduce harm. Furthermore, at a macro-policy level, we must actively resist “techno-solutionism”—the temptation for policymakers and health systems to deploy conversational AI as a cheap technological band-aid for profound structural deficits. A digital doula cannot, and should not, serve as an automated excuse for inadequate paid family leave, psychiatric workforce shortages, or the lack of fair compensation for human doulas and midwives.

The future of digital doulas should therefore be framed as a question of augmentation, not substitution. Their success will depend less on whether they can convincingly simulate supportive conversation and more on whether they can safely connect vulnerable patients to the right human care at the right time. In perinatal mental health, the central challenge is not simply building more intelligent tools; it is ensuring that these tools strengthen, rather than erode, the human relationships on which good care depends.

## Data Availability

The original contributions presented in the study are included in the article/supplementary material. Further inquiries can be directed to the corresponding author.
